# Gene polymorphisms of superoxide dismutases and catalase in diabetes mellitus

**DOI:** 10.1186/1471-2350-9-30

**Published:** 2008-04-21

**Authors:** Milan Flekac, Jan Skrha, Jirina Hilgertova, Zdena Lacinova, Marcela Jarolimkova

**Affiliations:** 1 3rd Dept. of Internal Medicine, 1st Faculty of Medicine, Charles University, Prague, Czech Republic

## Abstract

**Background:**

Reactive oxygen species generated by hyperglycaemia modify structure and function of lipids, proteins and other molecules taking part in chronic vascular changes in diabetes mellitus (DM). Low activity of scavenger enzymes has been observed in patients with DM. Protective role of scavenger enzymes may be deteriorated by oxidative stress. This study was undertaken to investigate the association between gene polymorphisms of selected antioxidant enzymes and vascular complications of DM.

**Results:**

Significant differences in allele and genotype distribution among T1DM, T2DM and control persons were found in SOD1 and SOD2 genes but not in CAT gene (p < 0,01). Serum SOD activity was significantly decreased in T1DM and T2DM subjects compared to the control subjects (p < 0,05). SOD1 and SOD2 polymorphisms may affect SOD activity. Serum SOD activity was higher in CC than in TT genotype of SOD2 gene (p < 0,05) and higher in AA than in CC genotype of SOD1 gene (p < 0,05). Better diabetes control was found in patients with CC than with TT genotype of SOD2 gene. Significantly different allele and genotype frequencies of SOD2 gene polymorphism were found among diabetic patients with macroangiopathy and those without it. No difference was associated with microangiopathy in all studied genes.

**Conclusion:**

The results of our study demonstrate that oxidative stress in DM can be accelerated not only due to increased production of ROS caused by hyperglycaemia but also by reduced ability of antioxidant defense system caused at least partly by SNPs of some scavenger enzymes.

## Background

It is a well-estabilished fact that diabetes mellitus is a risk factor for cardiovascular disease [1] which is leading cause of death in diabetic population [2]. It is also well-known fact that tight control of diabetes is effective in reducing vascular complications [3]. One of the principal pathways to develop vascular complications is the production of ROS [4]. There are multiple sources of oxidative stress in diabetes including nonenzymatic and enzymatic pathways [5]. While ROS are generated under physiological conditions [6], excess generation of ROS has pathological consequences [7]. ROS can stimulate oxidation of LDL, forming ox-LDL, which is not recognized by the LDL receptor leading to foam cell formation[8]. ROS can activate formation of AGE [9], polyol pathway [10], hexosamine pathway and PKC [11], involved in the pathogenesis of vascular complications [12]. Superoxide is dismutated to H_2_O_2 _by MnSOD in the mitochondria and by CuZnSOD in the cytosol [13]. H_2_O_2 _is converted to H_2_O and O_2 _by glutathione peroxidase or catalase in the mitochondria and the lysosomes [14].

We still have to elucidate the fact that some patients with DM develope vascular complications but this cannot be seen in the others with the same level of disease control [15]. We have focused on the genes encoding superoxide dismutases and catalase. Specifically, we have focused on SNPs for their likely functional role: SOD1 +35A/C (refSNP ID: rs2234694) which is located adjacent to the splice site (exon3/intron3 boundary), SOD2 Ala16Val (refSNP ID: rs4880) which has been suggested to alter protein structure [16] and function (C/T substitution in exon 2, codon position 2, aminoacid position 16) and catalase -21A/T (refSNP ID: rs7943316) which is located inside the promotor region just proximal to the start site.

CuZnSOD also called SOD1, EC 1.15.1.1 is one of the cellular defense systems for oxidative insults [17]. The increase of CuZnSOD expression in human smooth muscle cells protects against oxidative injury. OxLDL caused an increase in the DNA binding activity of activator protein-1 and nuclear factor κB, which is inhibited by CuZn-SOD overexpression. MnSOD also called SOD2, EC 1.15.1.1 is present in the mitochondria. C/T substitution (GCT/GTT) has been shown to change the structural conformation of the mitochondrial targeting sequence (MTS) of the enzyme. Associations have been found between the Ala16Val SNP and neurodegenerative disorders [18]. CAT, EC 1.11.1.6 is present in the peroxisomes and exists as a dumbbell-shaped tetramer of four identical subunits. Several SNPs in the CAT gene have been reported, most of these are associated with acatalasaemia [19].

## Methods

### Subjects

Total of 120 T1DM, 306 T2DM and control group of 140 healthy subjects without family history of diabetes were examined in this study. Diagnosis of T1DM and T2DM was based on WHO/ADA definition of diabetes (1999), healthy subjects don't meet the criteria for the diagnosis of DM. They were in good health and namely free of any comorbidities often associated with DM, especially with T2DM (arterial hypertension, obesity, hyperlipoproteinaemia) and other endocrine disorders. Microangiopathy was confirmed by ophthalmoscopy or by the presence of peripheral neuropathy (diagnosis was based on clinical features and by physical examination using 10 g monofilament, tuning fork and biothesiometry) in 167 patients who did not have any evidence of macrovascular disease from the clinical picture (no history of angina pectoris, normal ECG records or normal coronarography). Patients were excluded from this group in the case of suspection on autonomic neuropathy made from physical examination (tachycardia recorded by ECG in the resting state, systolic blood pressure reaction on orthostatism). 66 subjects had macrovascular complications manifested by ischaemic heart disease (diagnosis was based on ECG or coronarography), ischemic disease of the lower limbs (diagnosis was based on angiography of lower limbs arteries) or had history of stroke (diagnosis was based on clinical features and computer tomography). The remaining 161 diabetic patients were free of any complications. Clinical and laboratory characteristics are shown in Tab. 1. The research has been carried out within the ethical framework, informed consents of all participants are documented.

**Table 1:** Clinical and laboratory characteristics.

	T1DM	P values (a)	T2DM	Controls	P values (b)
Gender (males/females)	58/62	0,602	156/150	93/87	0,198
Mean age (years)	40 ± 12	**0,022**	57 ± 15	39 ± 9	**0,043**
Duration of DM (years)	18 ± 9	0,853	17 ± 8	0	-
BMI (kg/m2)	22 ± 4	**0,03**	30 ± 5	21 ± 5	**0,027**
Systolic BP (mmHg)	120 ± 10	0,748	125 ± 25	120 ± 20	0,676
Diastolic BP (mmHg)	60 ± 20	0,521	70 ± 30	70 ± 15	0,621
Microvascular complications(n)	40	**0,048**	159	0	-
Macrovascular complications(n)	14	**0,016**	52	0	-
FPG (mmol/l)	6,60 ± 1,35	0,058	7,82 ± 2,29	4,95 ± 0,76	**0,009**
HbA1c (%)	6,1 ± 1,9	0,321	6,7 ± 1,8	0	-
GFR (MDRD) (ml/s/1,73 m ^2^)	1,23 ± 0,35	0,179	1,09 ± 0,28	1,35 ± 0,22	**0,041**
Total cholesterol (mmol/l)	4,8 ± 0,5	**0,042**	5,3 ± 0,7	4,8 ± 0,3	0,205
HDL-C (mmol/l)	1,55 ± 0,35	0,216	1,31 ± 0,32	1,75 ± 0,66	0,325
LDL-C (mmol/l)	3,22 ± 0,53	0,116	3,57 ± 0,81	3,10 ± 0,74	0,234
Triglycerides (mmol/l)	1,31 ± 0,30	**0,031**	1,99 ± 0,79	1,23 ± 0,48	0,038

### Laboratory measurements

Venous blood samples were drawn after an overnight fasting. Plasma (Li-Heparine) glucose, creatinine were measured in central biochemistry laboratory. Serum total cholesterol, HDL-cholesterol and triglycerides (TG) were measured by automated enzymatic methods on Hitachi analyzer, LDL cholestrol was calculated according to Friedewald formula. HbA1c was measured by high-performance liquid chromatography. Superoxide dismutase activity was determined spectrophotometrically by xanthine/xanthine oxidase system by Genesys 5 spectrophotometer, USA. The method is based on the reaction described by McCord and Fridovich [20]. SOD activity was expressed in international units (U).

### DNA analysis

Blood was extracted from the peripheral blood (5–10 ml) and genomic DNA was prepared from leucocytes (minimal amount of leucocytes was 3,5. 10^9 ^/l) by sodium dodecylsulphate (SDS) lysis by ammonium acetate extraction and ethanol precipitation. Determination of the SOD and CAT polymorphisms was achieved by PCR-RFLP analysis. Details are shown in Tab. 2. Digested PCR products were visualised by UV transillumination following ethidium bromide staining and migration compared against DNA ladder and a positive RFLP control sample. 3% agarose gel including 0,5 μg/ml ethidium bromide, 10 μl of molecular markers (two different types used simultaneously) and 20 μl of amplicon for the other wells were applied for electrophoresis. 0,5 × TBE buffer (pH 8) including 0,5 μl/ml ethidium bromide was used. Running conditions were 100 V, 40 mA and 140 min. Informations about all SNPs and SNP ID were obtained from the NCBI homepage and all SNPs have been validated by multiple, independent submissions to the refSNP cluster. The genotyping success rate was 95.0% (range 91.1 to 98.4%). Water control, internal controls and previously genotyped samples were included in each plate to ensure accuracy of genotyping. Positive and negative controls were used in each genotyping assay. To ensure quality control, the genotyping analysis was performed "blind" with respect to case/control status. About 10% of the samples were randomly selected to be genotyped again by a different investigator, who was also unaware of the status of studied subjects.The results were concordant. The polymorphisms were also examined by PCR and RFLP analysis described previously [21-23].

**Table 2:** Sequences of used primers and used restrictases.

**SNP**	**sequence of used primers**	**restriction endonuclease**	**restriction fragments**
SOD1 35 A/C	5'CTATCCAGAAAACACGGTGGGCC 3'	HhaI	C allele 71 bp and 207 bp A allele 278 bp
	5'TCTATATTCAATCAAATGCTACAAAAC3'		
SOD2 A16V (C/T)	5'GCTGTGCTTTCTCGTCTTCAG 3'	BsaWI	C allele 267 bp T allele 183 bp and 84 bp
	5'TGGTACTTCTCCTCGGTGACG3'		
CAT -21 A/T	5'-AATCAGAAGGCAGTCCTCCC-3'	HinfI	A allele 203 bp and 47 bp T allele 250 bp
	5'-TCGGGGAGCACAGAGTGTAC-3'		

### Statistical analysis

Age, BMI and duration of diabetes were compared between studied groups using Student's *t*-test. Statistical analyses of frequency counts were performed using the Chi-square (χ^2^) test. Comparison of continuous variables (HbA_1c_) among the SOD genotypes was performed with the use of analysis of variance (ANOVA). A logistic regression analysis was performed to evaluate the interaction between the genotypes and other variables in relation to the prevalence of macro- or microangiopathy. In this analysis, the dependent variable was the presence or absence of vascular complication. Independent variables included in this analysis were BMI, age, present HbA_1c _level, type of diabetes, duration of diabetes, SOD activity and genotype. P values < 0.05 were considered as significant. The laboratory data are expressed as means ± SD. The analysis was performed using programme Statistica 6.0 (StatSoft). Testing for deviation from Hardy-Weinberg equilibrium (HWE) was performed and all the observed genotype frequencies were in agreement with HWE.

## Results

### SOD activity

Serum SOD activity was significantly decreased in T1DM (0,75 ± 0,18 U; 95% CI: 0,72, 0,79) and in T2DM patients (0,71 ± 0,33; 95% CI: 0,67, 0,74) compared to the control subjects (1,67 ± 0,33, 95% CI: 1,61, 1,72), both p < 0,01. Differences between T1DM and T2DM in SOD activity were not found statistically significant (p = 0,14). No gender or age influence on its activity was found in diabetic patients or healthy subjects. Difference in SOD activity between diabetic patients and healthy subjects is probably accountable not only by genotype background but also by various effects in terms of diabetes, e.g. enzyme glycation.The lower serum SOD activity was found in patients (T1DM and T2DM) with macrovascular complications (0,51 ± 0,31 U; 95%CI: 0,43, 0,58) than in those with microvascular complications (0,74 ± 0,16 U; 95%CI: 0,71, 0,76), p < 0,01 or without any vascular complications (0,76 ± 0,34 U;95%CI: 0,71, 0,81), p < 0,05.

### The effect of the SOD1 +35A/C polymorphism on SOD activity in healthy subjects and diabetic patients with DM

The AA genotype was the most common observed in the healthy subjects followed by the AC genotype, whereas the AC was more common than the AA genotype in T1DM and T2DM patients (Tab. 3). Significant differences between the allele and genotype frequencies for the SOD1 +35A/C polymorphism was observed in T1DM as compared to controls (A: 0.69 vs 0.52, p < 0.01; C: 0.31 vs. 0.48, p < 0.05) and similarly in T2DM (A: 0.58 vs. 0.52; C: 0.42 vs 0.48, p < 0.05). This SNP was related to SOD serum activity. Higher activities were found in AA than in CC genotypes of diabetic patients (Tab. 3). Statistical analysis (analysis of variance) showed significant trend towards possible association of AA genotype with higher activity (P (trend) = 0.029). Diabetic and healthy subjects have been pooled together as one group in the study of association between SOD activity and genotypes to improve statistical power of analysis. Differences among these subjects in age, duration of diabetes, presence of other co-morbidities were included.

**Table 3:** SOD activities according to genotype, genotypes frequencies.

		**AA**	**AC**	**CC**	**TT (Val/Val)**	**CT (Ala/Val)**	**CC (Ala/Ala)**	**AA**	**AT**	**TT**
**T1DM**	**n (%)**	58 (48)	50 (42)	12 (10)	79 (66)	36 (30)	5 (4)	48(40)	53 (44)	19(16)
	**SOD act (U)**	0,76 ± 0,15	0,74 ± 0,13	0,73 ± 0,19	0,73 ± 0,14	0,74 ± 0,23	0,77 ± 0,18			
**T2DM**	**n (%)**	104 (34)	147(48)	55 (18)	220 (72)	80 (26)	6 (2)	110 (36)	147 (48)	49 (16)
	**SOD act (U)**	0,73 ± 0,29	0,72 ± 0,36	0,70 ± 0,31	0,72 ± 0,28	0,72 ± 0,19	0,75 ± 0,15			
**Controls**	**n (%)**	49 (27)	90 (50)	41 (23)	52 (29)	90 (50)	38 (21)	67 (37)	86 (48)	27 (15)
	**SOD act (U)**	1,66 ± 0,31	1,67 ± 0,35	1,66 ± 0,28	1,58 ± 0,28	1,60 ± 0,35	1,63 ± 0,08			

The occurrence of genotypes in studied polymorphisms among T1, T2 diabetic patiens and healthy subjects (controls), serum superoxide dismutase activity separated according to the genotypes in compared groups (AA/AC/CC in SOD1 gene, TT/CT/CC in SOD2 gene, AA/AT/TT in CAT gene). Data are expressed in mean ± SD, *n *means number of cases, *brackets *mean genotype frequencies (allele frequencies and differences among them are mentioned in text), *SOD act *means enzyme activity.

### Relationship between the SOD2 Ala16Val (C/T) polymorphism and SOD activity in healthy subjects and patients with DM

**Figure 1:**
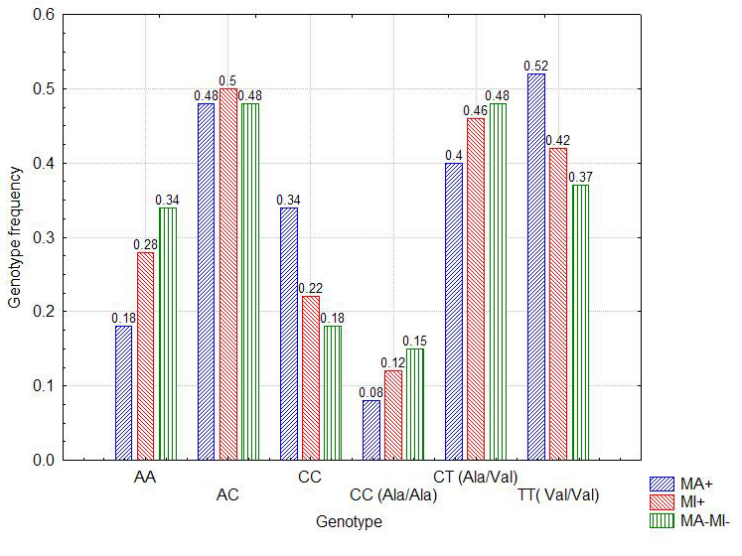
**Genotype frequencies according to presence of vascular complications**. Distribution of genotypes in SOD1 (A/C allele) and SOD2 (C/T allele, Ala/Val) in both types of diabetes mellitus according to presence of macroangiopathy (MA+) or microangiopathy (MI+) or no complications (MA-MI-). Explanation of results is mentioned in the text.

The TT genotype (Val/Val) was the most common in both T1DM and T2DM patients, CT genotype was the most common in healthy subjects whereas the CC genotype (Ala/Ala) was the rarest one in all groups (Tab. 3). The allele frequency of the SOD2 polymorphisms was significantly different in healthy persons compared to T1DM and T2DM patients (T allele (Val): 0.54 (controls) vs. 0.81 (T1) or 0.85 (T2), p < 0.05 and C allele (Ala): 0.46 (controls) vs. 0.19 (T1) or 0.15 (T2), p < 0.05 (Fig. 1). In all groups of diabetic patients SOD activity was the highest in the CC genotype (Ala/Ala) and the lowest in the TT genotype (Val/Val) (Tab. 3.).

### CAT polymorphism in diabetes mellitus

We found no statistically significant differences between frequencies in alleles of CAT SNP between diabetic patients and healthy subjects (p = 0,294) (Tab. 3). Control of diabetes was not influenced by polymorphisms in the CAT gene (Tab. 4).

**Table 4:** Genotype frequencies in CAT gene according to presence of vascular complications and values of glycated haemoglobin according to the genotype.

Genotype	MA+	MI+	MA-MI-	HbA1c
AA	0,28	0,32	0,34	6,28 ± 1,25
AT	0,50	0,49	0,49	6,35 ± 1,10
TT	0,22	0,19	0,17	6,32 ± 1,22

Genotype frequencies of CAT gene according to the presence of vascular complications in patients with diabetes mellitus. *MA+ *means presence of macroangiopathy, *MI+ *means presence of microangiopathy, *MA-MI- *group involves patients without vascular complications. *HbA1c *is glycated haemoglobin (%) marked as mean ± SD.

### The association of enzyme activity and polymorphisms in SOD1 and SOD2 with diabetes control and vascular complications of diabetes mellitus

Diabetes control expressed by glycated haemoglobin values was poorer in TT genotype (Val/Val) of SOD2 (7,10 ± 1,51; 95%CI: 6,42–7,91 in T1DM and 7,29 ± 1,49; 95%CI: 6,70–8,46 in T2DM) than in CC genotype (Ala/Ala) of SOD2 (6,39 ± 1,1; 95%CI: 5,7–7,0 in T1DM and 6,71 ± 1,21; 95%CI: 5,73–6,99 in T2DM), p < 0,05.

No effect of SNP in SOD1 gene on diabetes control was found. Glycated haemoglobin was 6,7 ± 1,4; 95%CI: 5,41–6,23 in T1DM and 6,9 ± 1,4; 95%CI: 5,48–6,09 in T2DM, both in AA genotype of SOD1 gene and 6,59 ± 1,22, 95%CI: 5,08–6,24 in T1DM and 6,61 ± 1,51; 95%CI: 5,02–6,05 in T2DM, both in CC genotype of SOD1 gene, with p = 0,124.

Similar findings were made in CAT gene. Glycated haemoglobin was 6,14 ± 1,11; 95% CI: 6,06–6,57 in T1DM and 6,50 ± 0,85; 95%CI: 6,32–7,25 in T2DM, both in AA genotype of CAT and 6,19 ± 1,32, 95% CI: 6,09–6,60 in T1DM and 6,61 ± 0,54; 95%CI: 6,45–7,05 in T2DM, both in TT genotype of CAT gene, with p = 0,249.

Significantly different genotype frequencies of SNPs were found in diabetic patiens (T1DM and T2DM) with macroangiopathy (MA+) in SOD1 and SOD2 genes. When compared these with CC genotype vs. AC and AA genotypes of SOD1: OR (odds ratio) was 1.73; 95% CI 1.45–5.37 with p < 0,05, CC genotype (Ala/Ala) vs. CT (Ala/Val) and TT (Val/Val) genotypes of SOD2: OR was 0,62; 95%CI 0,58–0,90 with p < 0,01. When compared AA genotype vs. AT and TT genotypes of CAT: OR was 1.05; 95%CI 0,78–1,13, p = 0,851.

Macroangiopathy was associated with significantly higher frequency of C allele in SOD1 gene (0,58 in MA group vs. 0,42 in DM group without complications, p < 0,01), lower frequency of C alelle (Ala) in SOD2 gene (0,28 in MA group vs. 0,39 in DM group without vascular complications, p < 0,05) whereas no such distribution was found in CAT gene, p = 0,594.

No differences in genotype frequencies were associated with microangiopathy (MI+). When compared these with CC genotype vs. AC and AA genotypes in SOD1: OR was 0,91; 95%CI 0.74–1.32 with p = 0.783, CC genotype (Ala/Ala) vs. CT (Ala/Val) and TT (Val/Val) genotypes in SOD2: OR was 0,96; 95% CI 0.52–1.38 with p = 0.852 and AA genotype vs. AT and TT genotypes in CAT: OR 1,04 95%; CI 0,37–1,26 with p = 0,814. No statistically significant differences in allele frequencies were found in all SNPs of the studied genes in the patients with microangiopathy when compared with patients without vascular complications (C allele in SOD1 was 0,47 in MI group vs. 0,42 in DM group without complications, p = 0,118, T allele in SOD2 was 0,35 in MI group vs. 0,39 in DM group without complications, p = 0,242). Frequencies of genotypes ranged according to presence of vascular complications in both types of diabetes mellitus are showed in Fig. 1.

**Figure 2:**
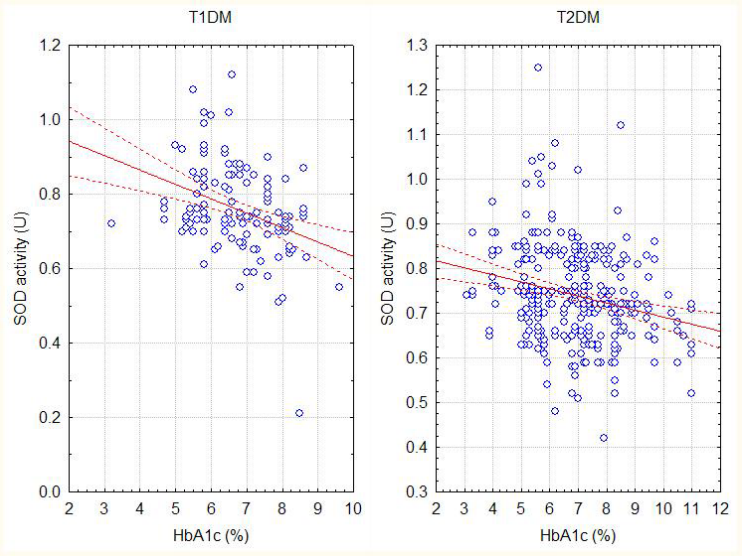
**Correlation between SOD activity and glycated haemoglobin**. Data correlation between the values of glycated haemoglobin (HbA1c %) and serum superoxide dismutase activity (SOD) in both types of diabetes mellitus. The correlation coeficients (Spearman) are r1 = -0,41 (T1DM), r2 = -0,23 (T2DM) with p < 0,05. Dotted lines mean 95% confidence intervals.

**Figure 3:**
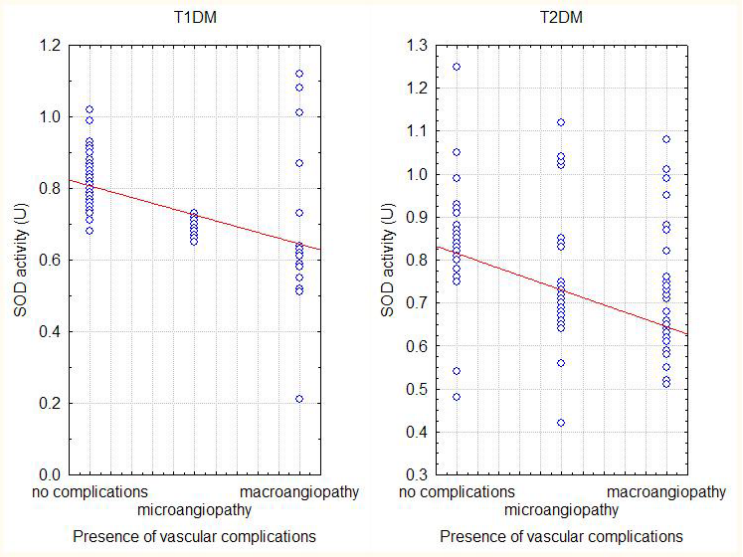
**Correlation between presence of vascular complications and SOD activity**. Cross-correlation between the presence of vascular complications in diabetic patients and the level of serum superoxide dismutase activity. The correlation coeficients (Spearman) are r1 = -0,29 (T1DM), r2 = -0,28 (T2DM) with p < 0,05. Dotted lines mean 95% confidence intervals.

We found negative correlation between serum superoxide dismutase activity (SOD) in both types of diabetes mellitus and the values of glycated haemoglobin (HbA1c %) (Fig. 2), as well as the presence of vascular complications in both types of diabetes (Fig.3).

Association of the SOD1, SOD2 and CAT polymorphism, BMI, age, duration of diabetes, sex, type of diabetes and SOD activity as independent variables with the presence of microangiopathy or macroangiopathy as dependent variables was performed using a logistic regression model. This analysis indicated that SOD1 and SOD2 genotypes are significantly associated with macroangiopathy. Another variables significantly associated (p < 0,05) with angiopathy were HbA1c, SOD activity and duration of diabetes. No independent contribution has been demonstrated for age, sex, BMI and the type of diabetes. (Tab. 5).

**Table 5:** Logistic regression analysis for risk factors of the vascular complications in diabetes mellitus.

Variable	p (MA)	OR; 95%CI (MA)	p (MI)	OR; 95%CI (MI)
SOD1 35 A/C	**0,048**	1,73; 1,45–5,37	0,783	0,91; 0,74–1,32
SOD2 A16V	**0,009**	0,62; 0,58–0,90	0,852	0,96; 0.52–1.38
CAT -21 A/T	0,851	1,05; 0,78–1,13	0,814	1,04; 0,37–1,26
SOD activity	**0,040**	0,48; 0,20–5,9	**0,048**	0,62; 0,44–0,91
Present HbA1c	**0,039**	1,28; 1,12–1,87	**0,041**	1,26; 1,12–1,81
BMI	0,686	0,97; 0,90–1,11	0,852	0,98; 0,87–1,04
Duration of diabetes	**0,038**	1,96; 1,38–3,27	**0,038**	2,01; 1,50–4,31
Sex	0,86	0,97; 0,89–1,22	0,80	0,95; 0,88–1,15
Age	0,124	0,73; 0,49–1,54	0,152	0,81; 0,52–1,28
Type of diabetes	0,084	0,96; 0,89–1,65	0,079	0,88; 0,73–1,17

*MA in brackets *means presence of macroangiopathy, *MI in brackets *means presence of microangiopathy, *OR *means odds ratio, *95%CI *means confidence interval (α = 0,05). MA and MI are dependent variables. Genotype (SOD1, SOD2, CAT), SOD activity, HbA1c, duration of diabetes, BMI, sex, age and type of diabetes act as independent variables. Variables significantly asssociated with macroangiopathy or microangiopathy are marked in table as bold.

## Discussion

Our findings in SOD2 gene are in agreement with previous observations of other authors [24]. We also confirmed known fact that serum SOD activity is significantly reduced in patients with DM [25]. The presence of TT (Val/Val) genotype in SOD2 gene was associated with poorer diabetes control in comparison with CT (Ala/Val) and CC (Ala/Ala) genotypes. Macroangiopathy was associated with significantly lower frequency of C (Ala) alelle of Ala16Val SNP of SOD2 gene. This has not been confirmed by another study focused on the role of antioxidative enzymes (including SOD2) in determining genetic susceptibility to the coronary artery disease in patients with T2DM [26]. Other studies suggest that Ala16Val SNP of SOD2 gene is not related to pathogenesis of diabetes but is associated with microangiopathy expressed as microalbuminuria [27] or macular edema in patients with T2DM [28]. No such distrubution was found in microangiopathy in our study. Finally, we found negative correlation between SOD activity in both types of diabetes and level of control (expressed by glycated haemoglobin) and presence of microangiopathy or macroangiopathy.

SNP in the signal sequence of SOD2 (Ala16Val) appears to be a minor determinant of carotid atherosclerosis [19]. The Ala type of SOD2 might have an common alfa-helical structure while the Val type might change its conformation to beta-sheet [29]. The Val variant of the SOD2 might be present at a lower concentration in the mitochondria. The processing study of these 2 leader signals has suggested that the basal level of the SOD2 activity might be the highest for Ala/Ala genotype (C/C) [29]. Observed positive association of macroangiopathy and high levels of glycated haemoglobin with the SOD2-Val/Val genotype could be explained, at least in part, by the Val izoform of the SOD2. It may lead to decrease resistance against ROS produced in the mitochondria and to oxidative damage of proteins[30]. The Ala allele of the SOD2 gene is more widespread than the Val allele in Caucasian population in contrast to Asian populations.

Our results are indicative of potential effect of A/C SNP in SOD1 gene on enzyme activity. It is known that deficiency in SOD1 results in increased levels of vascular superoxide and peroxynitrite and impaired endothelium-dependent relaxation in both large arteries and microvessels [31] and caused hypertrophy of arteries [32]. Macroangiopathy was associated with significantly higher frequency of C allele of +35 A/C SNP of SOD1 gene whereas no such distrubution was found in microangiopathy.

We found no statistically significant differences in distribution of CAT alleles in studied SNP and no impact of this SNP on the presence of vascular complications and the level of glycated haemoglobin. Hypocatalasaemic patients were found to have higher plasma levels of homocysteine and lower levels of folate [19], suggesting these patients are at greater risk for cardiovascular diseases. SNPs in the catalase promoter have been identified in a Swedish population [33] but their relationship to the vascular disease risk has not been determined. A variant within the catalase promoter region has been associated with essential arterial hypertension in an isolated Chinese population [34]. T1DM susceptibility locus on the chromosome 11p13 near to the catalase gene supports the idea of CAT gene may play a role in DM [35]. On the other side another authors found no evidence for a major effect of CAT SNPs on T1DM susceptibility in two large sample collections [36].

The study inconsistency in the association between genotypes and DM or cardiovascular disease is partly due to limits of conventional cross-sectional and retrospective case-control studies because selection bias have to be considered and the statistical analysis might have failed to demonstrate any significant differences. Large differences among ethnic populations are known in the distribution of genotypes which could be the reason for differences in studies.

## Conclusion

The presented findings show that the genotype distribution of the SOD1 and SOD2 in patients with diabetes mellitus can differed from nondiabetic individuals. We are conscious of the limitation of this study with relatively small sample size in comparison with wide epidemiological studies, especially by providing subgroup analysis within the group with diabetes mellitus. Nevertheless the results of small studies with similar conclusions may spark off suquent research. Genetic background may be at least partly associated with disease control of diabetes and consequently enzyme activities protecting against oxidative stress. Vascular disorders like atherosclerosis are then the results of combined genetic and metabolic changes.

## Abbreviations

AP-1: activated protein 1; AGE: advanced glycation endproducts; BMI: body mass index; CAT: catalase; JNK: c-Jun N- terminal kinase; CT: computer tomography; 95% CI: confidence interval 95%; ECG: electrocardiography; ERK: 1/2 extracellular signal regulated kinase; HbA1c: glycated haemoglobin; HDL: high density lipoprotein; LDL: low density lipoprotein; NFκB: nuclear factor kappa B; PKC: proteinkinase C; ROS: reactive oxygen species; RFLP: restriction fragment lenght polymorphism; SOD: superoxide dismutase; TG: triacylglycerol; T1DM: Type 1 diabetes mellitus; T2DM: Type 2 diabetes mellitus.

## Competing interests

The author(s) declares that they have no competing interests.

## Authors' contributions

MF drafted the manuscript, participated in design of the study, performed the statistical analysis, carried out the molecular genetic, participated in the sequence alignment. JS conceived of the study, participated in its design and coordination and helped to draft the manuscript. JH carried out the enzyme activities assessment. ZL carried out the molecular genetic, participated in the sequence alignment. MJ helped with the coordination of the study, participated in the genetic part of the study. All authors read and approved the final manuscript.

## Pre-publication history

The pre-publication history for this paper can be accessed here:


